# Participation in primary health care through community-level health committees in Sub-Saharan Africa: a qualitative synthesis

**DOI:** 10.1186/s12889-022-12730-y

**Published:** 2022-02-19

**Authors:** Robinson Karuga, Maryse Kok, Marthe Luitjens, Patrick Mbindyo, Jacqueline E. W. Broerse, Marjolein Dieleman

**Affiliations:** 1 LVCT Health, P.O. Box 19835, Nairobi, 00202 Kenya; 2 Athena Institute, Vrije Universiteit Amsterdam, De Boelelaan 1085, 1081 HV Amsterdam, The Netherlands; 3 KIT Royal Tropical Institute, Mauritskade 63, 1092 AD Amsterdam, Netherlands; 4 Jomo Kenyatta University of Agriculture and Technology, P.O. Box 62000, Nairobi, 00200 Kenya

**Keywords:** Community participation, Health committee, Community engagement, Social accountability

## Abstract

**Background:**

Health committees are key mechanisms for enabling participation of community members in decision-making on matters related to their health. This paper aims to establish an in-depth understanding of how community members participate in primary health care through health committees in sub-Saharan Africa (SSA).

**Methods:**

We searched peer-reviewed English articles published between 2010 and 2019 in MEDLINE, Popline and CINAHL databases. Articles were eligible if they involved health committees in SSA. Our search yielded 279 articles and 7 duplicates were removed. We further excluded 255 articles following a review of titles and abstracts by two authors. Seventeen abstracts were eligible for full text review. After reviewing the full-text, we further excluded two articles that did not explicitly describe the role of health committees in community participation. We therefore included 15 articles in this review. Two authors extracted data on how health committees contributed to community participation in SSA using a conceptual framework for assessing community participation in health. We derived our themes from five process indicators in this framework, namely, leadership, management and planning, resource mobilization from external sources, monitoring and evaluation and women involvement.

**Findings:**

We found that health committees work well in voicing communities’ concerns about the quality of care provided by health facility staff, day-to-day management of health facilities and mobilizing financial and non-financial resources for health activities and projects. Health committees held health workers accountable by monitoring absenteeism, quality of services and expenditures in health facilities. Health committees lacked legitimacy because selection procedures were often not transparent and participatory. Committee members were left out in planning and budgeting processes by health workers, who perceived them as insufficiently educated and trained to take part in planning. Most health committees were male-dominated, thus limiting participation by women.

**Conclusion:**

Health committees contribute to community participation through holding primary health workers accountable, voicing their communities’ concern and mobilizing resources for health activities and projects. Decision makers, health managers and advocates need to fundamentally rethink how health committees are selected, empowered and supported to implement their roles and responsibilities.

**Supplementary Information:**

The online version contains supplementary material available at 10.1186/s12889-022-12730-y.

## Background

Community-level health committees are defined as any formally constituted governance structures with community representation with an explicit link to a primary health facility. The primary purpose of these health committees is to enable participation of community members in decision-making on matters related to improving health service provision and health outcomes [1]. Many countries have health committees, which have the potential to be effective structures for active community participation in pursuit of universal health coverage by monitoring progress, identifying and solving problems and re-planning health priorities [2, 3]. Since health committees consist mostly of individuals from the community, they focus on quality services for their community and local health priorities. In response to regional and global calls for community participation in the management of primary health care, Ministries of Health in developing countries, in the early 1990s, established health committees to enhance participation of communities in the planning and development of primary health care and education programs [4]. Various forms of health committees exist. There are those health committees where lay community members participate in overseeing 1) the delivery of health services at household level, and 2) the management of primary health services in local primary health facilities.

Community participation in making oversight of primary health services contributes to improved health outcomes. A study by Loewenson et al. [5] in Zimbabwe demonstrated that health facilities that had a health committee had 1) more resources (financial and staff), 2) fewer drug stock outs, and 3) a higher coverage of primary health care services (6). In addition, the communities living within the area of coverage had better health knowledge and utilization of primary health services [5]. A study by Sohani et al. (2005) in Kenya demonstrated that health facilities with active health committees had better utilization of health services. This was associated with the health committees’ role in managing user fees, shaping local policy, recruiting and motivating community health workers (CHWs), providing healthcare education, establishing weekend outreach services for the remote villages, and increasing the availability of medicines [6].

Most countries in Sub-Saharan Africa (SSA) aspire to achieve universal access to healthcare for their citizens. Involvement of communities, through health committees, in the delivery of primary health services is essential for countries to achieve this aspiration [7, 8]. There is, however, limited knowledge on how health committees contribute to community participation in SSA [1]. Draper et al. [9] promulgated a conceptual framework to standardize the evaluation of community participation levels and processes in health programs using five process indicators: leadership, planning and management, women’s involvement, mobilization of external resources, and monitoring and evaluation. Since this framework focuses on different process indicators of community participation, it allows us to conduct a nuanced analysis of how health committees contribute to community participation [9]. The Draper et al. [9] framework proposes three levels of participation in the community participation continuum, and for each process indicator. The lowest level is “mobilisation”. At this level of the continuum, health professionals run primary health programmes and only mobilize community members for actions and to passively support decisions. The second level of participation is “collaboration”. In this level of participation, health professionals define primary health care needs of the community and invite them to contribute their personal resources and time in health promotion activities. The highest level of community participation in the continuum is referred to as “empowerment”, where community members have opportunities to exercise their power to make decisions that affect their health [9].

Operationalization and scale-up of community participation in health, including through health committees, is challenging and knowledge is rather fragmented. Therefore, increased insight into how health committees contribute to community participation will inform policy makers and health managers about the dynamics of community participation, through health committees, and how participation contributes to outcomes of community health programs [9]. This paper aims to establish an in-depth understanding of how community members participate in primary health care through community level health committees in SSA.

## Methods

We conducted a systematic review of qualitative studies in SSA to get an in-depth understanding of the features of health committees and how they contribute to community participation across a wide range of settings. Synthesis of qualitative evidence is helpful for interpreting and understanding subtle narratives on experiences and perceptions of actors involved in community participation within their contexts [10]. This section describes the procedures we followed.

### Search methods for studies in this review

In September 2019, we systematically searched for original peer-reviewed qualitative studies conducted in SSA. We included articles that applied mixed methods when they clearly described the qualitative research components. For studies to be eligible, they had to present an analysis of how health committees participated in health and had to be published in English between 2008 and 2019. We first searched the MEDLINE database using search terms that covered *population* (research conducted in SSA), *concept* (research related to community-level health committees), and *context* (primary health care settings in SSA) [11]. We then searched the Popline database before it was retired in September 2019 and CINAHL. The search terms are illustrated in Supplementary file 1.

### Selection of studies

**Fig. 1:**
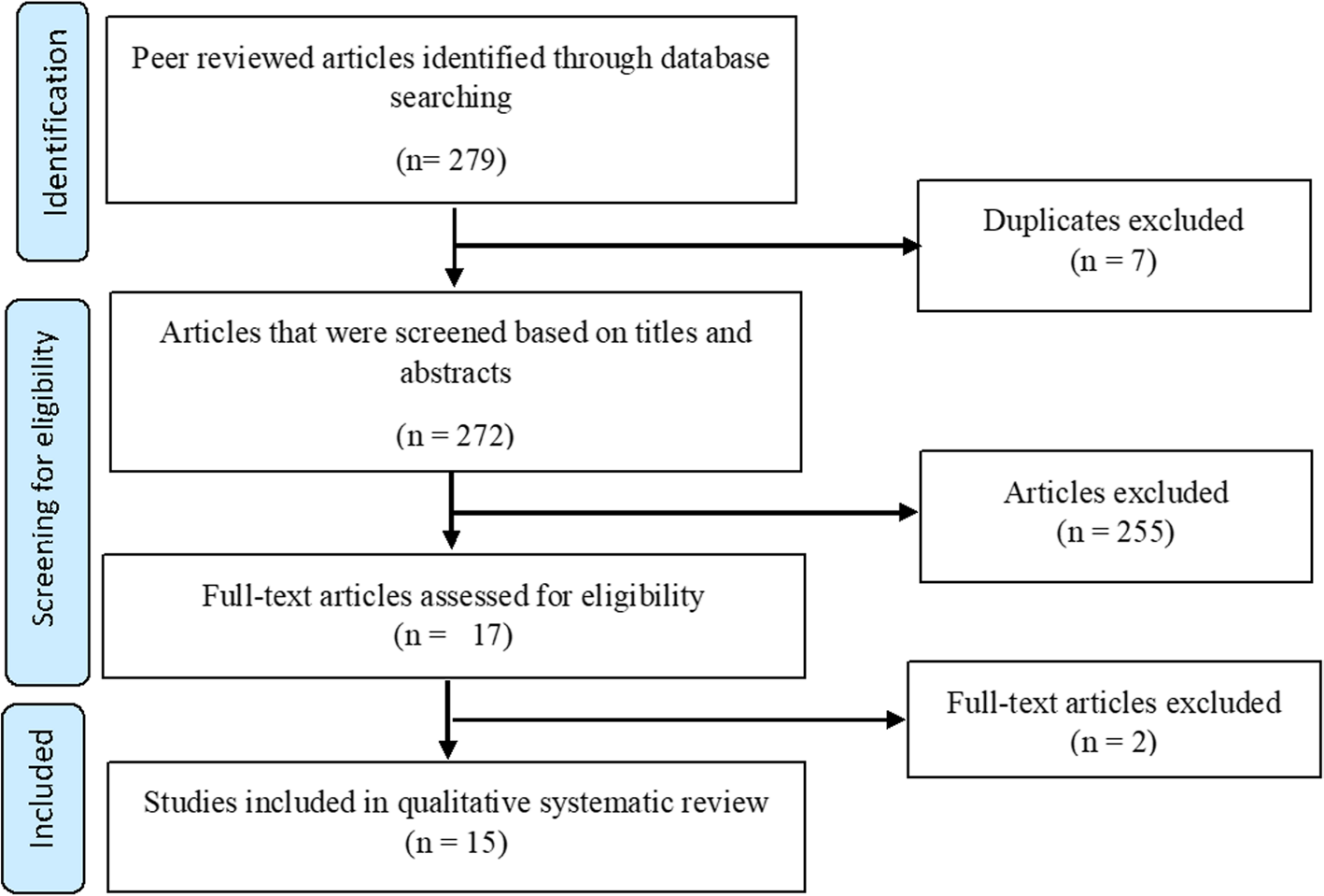
Flow Chart summarizing the process of selecting articles to include in this review

Our database search identified a total of 279 articles. One author uploaded all articles from the database searches on to the Rayyan Systematic Reviews Web App (https://rayyan.qcri.org/). Two authors (ML and RK) independently assessed the titles of articles to identify the ones that fit into this review by examining the subject and where the research was conducted. Seven duplicates were identified and deleted at this phase of screening. From the remaining 272, abstracts of articles that seemed relevant were read by two researchers to assess whether their research questions, study populations and methodologies aligned with the inclusion criteria of this review. As a result, we excluded 255 articles because they were not about health committees. ML and RK then examined the full text of the 17 articles that met the eligibility criteria for further screening. Any conflicts at each step of screening were discussed among the two authors until consensus was reached. We further excluded two articles that did not explicitly describe the role of health committees in community participation. Fifteen articles were included in this review. The flowchart in Fig. 1 summarizes the selection process. We then transferred all eligible articles into the EndNoteX7.5 reference management software.

### Data management, extraction and analysis

We began by extracting data on the first author, year of publication, study setting and a summary of the study from the identified papers. Two reviewers then conducted an in-depth data extraction on how health committees contributed to community participation in SSA using the conceptual framework proposed by Draper et al. [9] for assessing community participation in health. The two reviewers (RK and ML) independently read all the articles that were eligible for inclusion in this review. Any discordances between the reviewers during article selection and data extraction process were resolved during weekly analysis meetings. We derived our themes from the five process indicators in this framework, namely, leadership, management and planning, resource mobilization from external sources, monitoring and evaluation, and women involvement (Table 1). We deductively coded text from the research articles in the five themes using Microsoft Excel. Where extracted data seemed to fit in more than one theme, we agreed on the most relevant theme to fit it in. Three authors discussed the synthesis of evidence for one theme at a time.

**Table 1:** Definition of process indicators in our conceptual framework [9]

Themes	Definition of process indicators	Number of articles contributing to each theme
Leadership	The extent to which health committee members provide leadership in decision-making and how interests of various community groups are represented through health committees	10 [12–21]
Management and planning	The extent to which health committee members define priorities and manage community health services.	10 [13–15, 17, 20–24]
Resource mobilization from external sources	The extent to which health committee members find ways of mobilizing resources for running health-related activities at the community level.	6 [17, 20–23, 25]
Monitoring and evaluation	The extent to which health committee members conduct participatory evaluation of health services that produce local meaningful findings.	5 [16, 17, 20–23]
Women involvement	The extent to which women actively participate in decision-making through the health committee	4 [12, 13, 22, 26]

## Results

We start this section by providing an overview of the articles included in this review. We then describe how health committees contribute to community participation. Our review findings are organized based on the themes derived from the conceptual framework used during analysis.

Studies included in this review were from diverse geographical settings in SSA: nine studies were conducted in Eastern Africa (Kenya, Burundi, Uganda, and Tanzania); two were conducted in southern Africa (Malawi and Mozambique) and four were conducted in Western Africa (Nigeria and Sierra Leone), and one was a multi-country study that was conducted in Guinea, Benin and the Democratic republic of Congo (Table 2).

**Table 2:** Details of studies included in this review

Author	Country(ies)	Name Health Committee and roles	Study description	How included articles described selection of Health Committee members	Methods applied in the selected articles
**Lodenstein et al 2019** [21]	Malawi	**Health Centre Advisory Committee (HCAC)** **Expected roles and responsibilities** 1. Bridging the communication gap between community and primary health workers in facilities 2. Inspection of conditions and drug stock in primary health facilities 3. Formulating recommendations on facility equipment 4. Management of complaints from users of primary health providers	Study explored experiences with, and perceptions on, the role of HCACs as social accountability interfaces and the approaches HCACs use to address poor service quality and performance in rural health centres.	Although the selection of HCAC members is not described in this article, the Ministry of Health (MoH) guideline stipulates that members should be elected by community members.	Authors conducted • 62 individual interviews in 22 Health Centres (22 with HCAC members and 40 with primary health workers) • 7 key informant interviews with District Health Management Team members • Review of 12 HCAC Minutes • Review of written communication between HCACs and health workers in Mzimba North and South Districts in Malawi
**Ogbuabor and Onwujekwe 2018** [23]	Nigeria	**Health Facility Committee (HFC)** **Expected roles and responsibilities** 1. Monitor the delivery of free primary health care and identify eligible users for these services 2. Provide platforms for consultations between health workers and clients 3. Raise awareness about free primary health services 4. Mobilise communities to use public health facilities 5. Manage facility resources and facilitate implementation of the complaint systems 6. Joint problem analysis and planning with other stakeholders at the facility and policy levels	Study provided evidence on how social accountability initiatives influenced revenue generation, pooling and fund management, purchasing and capacity of health facilities implementing the free maternal and child healthcare program.	HFC members are selected to represent special groups by their communities for a renewable term of 3 years	Authors conducted: • Review of 14 policy documents • In-depth interviews (IDIs) with 44 participants (16 policymakers, 16 providers and 12 HFC leaders) • 4 focus group discussions (FGDs) with 42 women in Enugu State in South East Nigeria
**Lodenstein et al., 2017** [16]	Benin, Guinea and Democratic Republic of Congo (DRC)	**Benin: Health Facility Management Committee (COGECS)** **Guinea: Health Management Committee (COGES)** **DRC: Health Development Committee (CODESA)** **Expected roles and responsibilities** COGECS (Benin) 1. Monitoring of the primary facility budgeting process 2. Management of user fees generated in primary health care facilties 3. Establishment of drug inventories and orders 4. Promote financial transparency in pricing policies in primary health facilities 5. Resolution of conflicts community and primary health care providers COGES (Guinea) 1. Plan and monitor services in primary health facilities. 2. Supervision of health workers based in primary health facilities 3. Maintain dialogue between primary health providers and the community CODESA (DRC) Planning and monitoring health services in partnership with primary health care providers	Study explored the functioning of health committees in particular with regard to their actual and potential role in the facilitation of social accountability	HFCs in the three countries were constituted through elections. The article however reported diverse methods of reconstituting members after the elections.	Authors conducted 95 individual interviews and 22 focus group discussion (FGDs) in: 4 Districts in Benin, 4 Zones in DRC and 4 prefectures in Guinea.
**Iyanda & Akinyemi, 2017** [26]	Nigeria	**Village Development Committee (VDC)** **Expected roles and responsibilities** Serving as the interface between government and community in decision making, planning, execution, and analysis of development tasks at village level	Study assessed community participation as a major principle in the delivery of primary health care services	Selection of VDC members not described in this article	45 participants were enrolled for 12 in-depth interviews (IDIs) and 4 FGDs with primary health care providers and community members in Ibadan South East Local Government Area of Oyo state, Nigeria.
**McMahon et al., 2017** [25]	Sierra Leone	**Health management committee (HMC)** **Expected roles and responsibilities** HMCs on Sierra Leone do not have a standard structure and roles. They take on different forms, have different planned and actual roles, and receive support various NGOs and government programs.	Study described the roles played by members of HMCs and related forms of community-based voluntary engagement during the Ebola outbreak in two districts of Sierra Leone.	The process of constituting HMC was not described in this article	Authors analysed data from 13 FGDs with health committee members in 8 peripheral health units in urban and rural settings across Bo and Kenema districts, which represented areas of low and high Ebola transmission, respectively.
**Abimbola et al., 2016** [20]	Nigeria	**Community Health Committee (CHC)** **Expected roles and responsibilities** 1. Identify the health needs of the community, and address them by drawing on human and material resources within the community, including raising funds when necessary within the community; 2. Liaise with the government and NGOs in finding solution to health needs of the community 3. Supervise and support health activities in the community and at the health facility, including the drug revolving funds 4. Signatories to primary health facility bank accounts (chairman, treasurer and secretary)	Study conducted an in-depth exploration of how and under what circumstances community health committees function.	Primary health care managers and NGOs convene town hall meetings with community members where they community members to nominate members to the health committee in accordance with federal guidelines, and read out to committee members their expected roles and responsibilities	Authors reviewed 581 meeting minutes of 129 CHCs in Lagos, Kaduna, Benue and Nasarawa States of Nigeria using a realist synthesis approach.
**Turinawe et al 2015** [19]	Uganda	Village Health Team* (VHT) **Expected roles and responsibilities** Mobilizing communities to utilize primary health services, health promotion and education, follow up clients that utilize primary health services and management of community information systems.	Study examined the process used to introduce the VHT strategy and how team members were selected in one rural community. The study also examined how these processes may have influenced the work of VHTs	Selection of VHT members was not done in accordance with MoH guidelines by Local Councils.	This study utilized ethnographic approaches that entailed: • Participant observation provided a point of entry into the community through joining in the activities of daily life • Spontaneous interactions yielded insights into the lives of community members • Field notes were taken on a daily basis to keep track with common activities • 12 FGDs and 14 IDIs with community members • 4 key informant interviews
**Turinawe et al 2016** [18]	Uganda	Village Health Team* (VHT) Roles are described in the article by Turinawe et al. 2015 [19]	Study critically examined the interplay between health officials and the community in the selection of VHTs in Ugandan rural setting.	Selection process similar to Turinawe et al. 2015 [19]	Authors used ethnographic approaches to conduct: • Observation of community events • 35 IDIs • 20 FGDs • 15 informal conversations, and; Review of project documents in Luwero District in Uganda
**Maluka & Bukagile, 2016** [17]	Tanzania	**Health facility committees (health centre committees and dispensary committees)** **Expected roles and responsibilities** 1. Discuss and pass health centre plans and budgets 2. Identify and solicit financial resources for running health centre 3. Ensure delivery of healthcare services	Study examined the differences in practice of health facility committees in a well- functioning district and one that is not.	Health Committee members were selected by Village Executive Officers and Ward Executive Officers after applying to be members. The list of shortlisted members was submitted to the Ward Development Committee for final selection and then forwarded to the District council for endorsement.	Authors reviewed policy documents MoH guidelines, minutes, committee records, schedule of meetings) and conducted: • 83 interviews • 449 structured exit interviews with clients seeking outpatient services • Health facility visits to verify and validate key issues identified by respondents
**Capurchande et al., 2015** [12]	Mozambique	**Community Health Committee (CHC)** **Expected roles and responsibilities** 1. Promote the use of family planning services 2. Mobilising and counselling community members to take up family planning services in primary health care facilities	Study examined how CHCs implemented family planning promotion activities and the complexities involved in the triangle of health. Workers, committee members and clients	The process of constituting CHC was not described in this article	Authors conducted: 6 FGDs; 4 informal conversations and reviewed policy documents and literature focusing on family planning observations in Boane and Ndlavela Administrative regions, Mozambique.
**Kilewo & Frumence, 2015** [15]	Tanzania	**Health Facility Governing Committee (HFGC)** **Expected roles and responsibilities** 1. Develop plans and budget for primary health facilities 2. Mobilize local communities to contribute to community insurance funds (e.g. Community Health Fund) 3. Ensuring the availability of drugs and equipment in primary health facilities 4. Reporting health provider employment and training needs to the district council, and ensuring adequate staffing levels in local primary health facilities 5. Liaise with Dispensary Management Teams (DMT) and other actors to ensure the delivery of quality primary health services.	Study explored factors that hinder community participation in developing and implementing Comprehensive Council Health Plans.	Constituting HFCGs varied from one health facility to another. Some members were handpicked by health workers, while others were elected by community members.	Authors conducted IDIs and discussions with 18 key informants in Singida Region, Manyoni District, Tanzania.
**Frumence, Nyamhanga, Mwangu, & Hurtig, 2014** [22]	Tanzania	**Health Facility Governing Committee (HFGC)** Same as in Kilewo & Frumence, 2015 [15]	Study explored the challenges and benefits of the participation of HFGCs in health planning in the decentralised health system in Tanzania.	Formation of HFCGs not described in this paper. We assume that the process is similar to the process described in the article by Kilewo & Frumence, 2015 [15]	Authors conducted document reviews, 13 key informant interviews and 6 FGDs at different health system levels in Kongwa district, Tanzania.
**Falisse, Meessen, Ndayishimiye, & Bossuyt, 2012** [13]	Burundi	**Comité de Santé (COSA)** **Expected roles and responsibilities** 1. Facilitate relations between client population and primary health care providers 2. Technical co-management of the primary health facilities (planning and evaluation), 3. Administrative co-management of primary health facilities, including controlling the finances 4. Participate in health promotion activities in their communities	Study aimed to analyse the place of two downward accountability mechanisms in a performance-based financing scheme.	COSA members were elected by residents living in the catchment areas of a Health Centre	Semi-structured questionnaires were conducted combining a classical ‘community participation in health’ framework and interviews in 6 provinces of Burundi.
**Goodman, Opwora, Kabare, & Molyneux, 2011** [14]	Kenya	**Health Facility Committee (HFC)** **Expected roles and responsibilities** 1. Oversee the general operations and management of primary health facilities 2. Advise the community on matters related to the promotion of health services 3. Represent and articulate community interests on matters pertaining to health in local development forums 4. Facilitate a feedback process to the community pertaining to the operations and management of primary health facilities 5. Implement community decisions pertaining to their own health 6. Mobilise community resources towards the development of health services within their area	Study assessed the nature and depth of managerial engagement of HFCs at the facility level and how this has contributed to community accountability in the context of the Direct Facility Financing mechanism.	Health Facility Committee members were selected in two ways. First, HFC members were elected by community members living in the catchment area of the Health Centre. Secondly, Chair persons of Village Health Committees (now referred to as Community Health Committees) were automatically nominated to the HFC as members.	Authors implemented: • 30 Structured interviews with Health workers in charge of primary health facilities • 292 structured exit interviews with community members seeking outpatient services in primary health facilities • 7 IDIs with District health Managers • 6 group discussions with health workers in 7 districts within Kwale and Tana River Counties
**O’Meara et al 2011** [24]	Kenya	Health Facility Committee Same as in Goodman, Opwora, Kabare, & Molyneux, 2011 [14]	Study described the national guidelines for the 2008–2009 annual planning process and discussed how these guidelines were implemented in Kilifi County. The study also highlighted the strengths and weaknesses of the planning process with regard to capacity at the implementation level and the engagement of communities in setting priorities in health.	The process selecting HFMC members was not described in this article. We assume he process is same as the process described in Goodman, Opwora, Kabare, & Molyneux, 2011 [14]	Authors employed: • Structured observation of the annual work plan and budget development forums and processes • Review of national guidelines for the planning and budgeting process • In-depth review of all minutes produced during meetings of facility committees related to the planning process. • Analysis of targets from the work plans produced by each primary health facility during the planning process in Kilifi County

*MoH* Ministry of Health, *IDI* In-depth Interview, *FGD* Focus group discussion

### Leadership

We examined the extent to which health committees provided leadership in decision making and how they represented the interests of various community groups. Health committees were mainly involved in making decisions related to day-to-day management of primary health facilities. Two sub-themes emerged under the representation role of health committees. First, representation of various community groups in health committees was influenced by the way these committees were constituted. Second, we found that health committees represented their communities by voicing their concerns about the quality of service delivery.

#### Representation through selection of health committee members

Eight of the 15 studies in this review documented how the process of constituting health committees influenced their representation role. There were variations in the way health committees were constituted. They were either constituted through elections or nominated directly by health workers, District health managers and village leaders. In other settings, village leaders selected themselves and fellow elites in the village to be health committee members.

Election of health committee members was reported in three studies. There were however deviations from election norms and procedures that affected representativeness of these committees. Two studies in Kenya and Burundi reported lack of transparency in the election process. Goodman et al. [14] found that existing chairs of health committees, in the coastal part of Kenya, were automatically selected to continue being committee members. As a result of lacking transparency, these members got away with retaining their positions perpetually. Since the election of committee members was not clear to community members, the majority of them had never heard of health committees, their members and roles. The same study revealed that 80% of community members did not know how committee members were elected [14]. Falisse et al. [13] documented how health committees in some Burundian settings had never held elections. Some committee members had not been replaced for up to 7 years without being elected by the communities they were supposed to represent [13]. Lodenstein et al‘s [16] study on the role of health committees in social accountability in Guinea, DRC and Benin reported that communities elected committee members, but health committee chairpersons reconstituted committee membership immediately after the elections by dropping key members who they perceived as being either incompetent, “too old” or “too uneducated”. Committee chairpersons then invited new members into these committees without consulting other members. These actions resulted in internal opposition, tension and apathy among health committee members [16].

In other settings, health committee members were directly nominated without involvement of community members in elections. Two Tanzanian studies reported how government officials, village leaders and primary health workers appointed committee members. Ward-level Officers advertised vacancies in health committees and invited community members who met the eligibility criteria to apply. Applicants were required to be literate and have completed primary education, among others. Ward-level Officers shortlisted the applicants and did the final selection on behalf of District health managers. In most cases, village leaders and health facility in charges directly nominated persons they knew to be part of the health committees. This mode of constitution alienated health committee members from their constituents, who were not aware of the existence of health committees and their roles [15, 17]. The same was reported in Mozambique, where community health committee members were nominated by health workers and community leaders [12]. Two studies in Uganda showed how village leaders selected themselves and other elite community members into health committees without holding elections. Turinawe et al. (2015) used the term *“elite capture”* to describe how village council leaders in Uganda influenced the process for selecting Village Health Team (VHT) members. In their desire to wield more power and authority, village council leaders ignored Ministry of Health (MoH) guidelines on the composition of VHTs and appointed themselves, their friends and certain people with formal education without involving other community members. Village council leaders justified not holding elections to constitute VHTs because community members did not show up for such meetings. Two studies in Uganda showed that this mode of constituting VHTs resulted in frustration among community members, suspicion, mistrust, resentment and open hostility towards VHTs by communities [18, 19].

A notable observation was that primary health care workers and District health managers in Kenya and Uganda abetted malpractices in the constitution of health committee members. In Uganda, health workers who supervised the composition of VHTs were in favour of village council leaders conveniently selecting people they could “work with” into VHTs. They also did not persuade village council leaders to convene elections [18, 19]. Goodman et al. [14] also demonstrated how sceptical District health managers in Kenya were about the process of electing health committee members by the community. They perceived elected health committee members as “old”, illiterate and lacking capacity to manage a health facility. Some District health managers sensitized communities on the importance of electing elite persons in the community such as retired professionals. Election of elite persons in the health committees resulted in intimidation of the less educated committee members [14].

#### Representation by voicing community concerns about health service delivery

Three studies in Kenya, Malawi and Nigeria documented how health committees represented their communities by actively raising their concerns about the quality of health services to District health authorities. Committee members voiced community concerns about health facility staff that were either abusive, disrespectful or absconded duty, inflated the cost of drugs or failed to be transparent with financial records [14, 20, 21]. In Kenya and Malawi, health committees lobbied District health managers to replace and discipline facility health workers that were involved in misdemeanour. One study described how health committees lobbied district health authorities to provide additional health workers and support staff to facilities. They also lobbied District authorities against transferring of health workers that had a good working relationship with committees and for payment of unremitted health worker allowances and salaries [20].

### Management and planning

In this theme, we explored the extent to which health committee members were involved in managing health services, and defining priorities and budgeting.

#### Health committees’ role in day-to-day management of health facilities

Six studies conducted in Nigeria, Kenya and Tanzania identified the active role of health committees on the day-to-day decision making and management of primary health facilities. Five of the six studies reported that health committees made decisions regarding the purchase and management of drug stocks, and supervision of projects such as construction and rehabilitation of health facilities. In some cases, health committees made the final decision on the expenditure of user fees that were collected in their health facilities [15, 17, 20, 22, 23]. Two studies conducted in Kenya and Nigeria reported that health committees were responsible for employing and managing support staff (grounds men, security guards, cleaners) in their health facilities [14, 20]. Lodenstein et al’s multi-country study described how health committee members in Benin made health workers pledge that they would agree to provide services and charge drugs at set prices. They also made health workers to agree to the working conditions in the presence of District health managers. Health workers who failed to abide by their pledges were disciplined by either being transferred to other areas or dismissal by committee members [16]. Two studies reported improvements in cleanliness and sanitation within primary health facilities where health committees participated in day-to-day management [17, 22].

Abimbola et al. and Goodman et al. noted cases of conflict between health workers and committee members about management of health facility resources. Conflicts arose when health workers perceived that health committee members were micro-managing them or when committee members demanded for finances from the facility accounts because they perceived themselves as owners and “watch-dogs” of the health facilities [14, 20].

#### Health committees’ role in priority setting and budgeting

Despite health committee involvement in day-to-day management of health facilities, they were often left out in development of annual budgets and plans. Seven studies identified gaps in health committees’ participation in setting priorities and making decisions regarding the management of their facilities. In Burundi and Tanzania, health committee members were not aware about the existence of annual primary health plans and budgets. Health committee members also did not know they had a role in the development of these plans and budgets. Committee members reported that none of them had been trained on their management roles [13, 15, 17]. Two other studies conducted in Kenya revealed that health plans and budgets were either developed by facility health workers or by a few health committee officials who had attended the initial committee trainings when health committees were formed with support from a donor-funded project. Overall, health workers actively left out committee members in planning and budgeting for health activities and facilities, because they perceived these committee members as low educated and lacking capacity [14, 17, 22–24]. Three of the seven studies reported that health committees had not been trained on their roles and responsibilities in planning health activities and also lacked budgets to support their involvement in planning (e.g. transport reimbursements, honoraria). These factors affected the performance of health committees in management and planning for health activities and facilities [15, 21, 22].

### Resource mobilization

Overall, six studies documented the role that health committees played in mobilizing resources to support health care delivery through 1) donations and charging user fees, 2) organizing community members to contribute their time, skills and raw materials, and 3) lobbying District managers to post and retain health workers and support staff in their primary health facilities.

Four of the six studies that reported resource mobilization by health committees documented how committee members solicited financial donations from prominent persons in the community and among committee members to undertake projects in their health facilities (construction of wards and health worker accommodation) [21, 25]; clean the health facility; pay for utilities such as water, electricity, minor repairs and pay for meeting costs (transport allowances and refreshments); and employ support staff [17, 22]. Abimbola et al. (2016) observed that health committees mobilized financial resources hoping that the government would supplement their resource mobilization efforts by “topping up” what they collected [20]. There were mixed findings about health committees’ role in raising financial resources through user fees. In Goodman et al’s [14] study, some committees were not allowed to set user fees because only the MoH had that mandate, while others were free to set their own user fees. Health committees in Abimbola et al’s [20] study sold facility supplies that they had received from government and non-government sources to clients seeking primary health care. Committees used finances generated from these sales to pay for utilities (electricity, water), repair and pay themselves meeting allowances [20].

Health committees also mobilized non-financial resources to support health care delivery and projects. In two studies in Tanzania and Nigeria, health committee members organized community members to contribute their labour, time and skills for the construction of health facility infrastructure, such as wards and placenta-pits, and cleaning health facilities [22, 23].

### Monitoring and evaluation

Our assessment did not find studies that documented health committee involvement in participatory evaluation. Instead, health committee members monitored the quality of care by health workers, drug stocks and financial records in the facility.

Five articles identified health committees’ role in monitoring the quality of health care by health workers in two ways. First, health committees received complaints from community members about quality of services in the facilities, lateness and absenteeism by health workers, denial of care and patient abuse and reporting them to the local health authorities. In one Nigerian study health worker salaries were not paid until the chair of the health committee endorsed the “staff time book” to curtail health worker absenteeism [23]. In another study from Nigeria, health committees required health workers to display a duty roster and weekly schedule of activities on their notice board to ensure that clients knew when to expect what services [20]. Committee members collected information through direct observation of services, inspecting health facilities and interacting with health facility clients [16, 20, 21, 23]. Maluka et al. (2016) reported that health services improved after health committees disciplined abusive health workers by having them transferred [17].

Secondly, health committees actively monitored drug stocks delivered to facilities, supplies delivered for facility renovations and also checked the veracity of financial records [16, 20, 22]. Committee members would then report any malpractices for disciplinary action to either District authorities or non-government actors that were supporting health facility projects. Lodenstein et al. [16] reported that health committees in West African settings introduced regulations to make health workers who issued false bills to refund the monies obtained fraudulently back to the health facility. Failure to comply with these regulations led to transfers of health workers to other facilities [16].

### Women involvement

None of the studies in this review had an in-depth exploration of the extent to which women actively participated in decision-making through health committees. Instead, articles reported the gender composition – number of male and female members. Three studies reported that men dominated membership and leadership in health committees. These studies only focused on how inadequate women’s representation was [13, 22, 26]. It is only Capurchande et al*’*s (2015) study in Mozambique where most health committees had slightly more women than men [12].

## Discussion

We set out to synthesize qualitative evidence understand how community members in sub-Sahara Africa participate in primary health care services through health committees. This synthesis provides evidence on the dynamics of community participation in community health programs. In this section, we first provide a summary of the findings, followed by a discussion of the key review findings, some recommendations, and limitations of the study.

Overall, we found that health committees were involved in a number of areas such as: voicing their communities’ concerns about the quality of care provided by health facility staff; day-to-day management of health facilities; and mobilizing financial and non-financial resources for health activities and projects. Health committees held health workers accountable by monitoring absenteeism, quality of services and expenditures in health facilities. We identified several challenges that influenced their success. Firstly, health committees lacked legitimacy, because selection procedures were often not transparent and participatory. As a result, communities were not fully aware of the existence of committees and their roles. Secondly, committee members were often left out in planning and budgeting processes by health workers who perceived them as insufficiently educated and trained to take part in planning. Thirdly, most health committees were dominated by male members, which limited the participation by women in voicing their health priorities. Below, we explore two key factors that influence the operationalization of community participation through health committees. We discuss how power dynamics influence selection of committee members and their involvement in planning and budgeting. We then have an in-depth discussion on tokenism in health committees.

### Power dynamics in community participation

Consistent with existing literature, our review reveals two ways how power dynamics manifest in community participation [27]. First, primary health workers and health managers use their power to influence the composition of health committees. We saw, in a number of settings, that health workers select or influence the election of elite and educated persons to these committees. This modification of selection procedures leads to elite capture and presents opportunities for individuals or elite groups to advance their own interests. Elite capture affects representativeness of health committees, which denies health committees impartiality, public spirit and support that is much needed for community participation [28]. Modification of selection processes without transparent involvement of community members also creates an environment where health committees lack legitimacy and are alienated from their constituents [29, 30]. Elite members may not understand the needs of their constituents or may not have similar ethnic or social-economic status as those they represent [31, 32]. Interestingly, elite members in health committees did lobby district authorities for disciplinary action when primary health workers were abusive or absent, mobilize resources to improve facilities and monitor absenteeism among health workers. It is possible that concerns voiced were those of elite individuals in the community. This was probably because they feel empowered to challenge or talk with those in authority. It is however not clear whether this sense of empowerment could enable them to address issues of importance to the broader community. On the other hand, having a more representative group in the committees might lead to a committee that is less “respected” by local authorities, and less listened-to by health workers as they are not “educated enough”.

Second, primary health workers and managers manifest power by dominating and controlling the planning and budgeting processes [27]. This is despite primary health workers and health managers favouring the selection of elites into the committees. Leaving out committee members during planning and budgeting processes may be a form of resistance by health workers and managers against community participation. Engaging lay individuals in developing health facility plans, albeit them being elites, is a shift from the traditional physician-dominated, biomedical illness-care system and tensions arise when health workers feel threatened by potential reduction of influence and control to lay committee members [31]. Previous research shows that health professionals tend to place more importance to formal education and specialized skills and undervalue local knowledge possessed by health committee members, despite some of them being relatively more educated. While technical expertise is needed in management of community health, it must be accompanied by local knowledge (history, culture, gender and power relations, terrain, geo-politics) for it to suit the communities’ needs [33]. Health workers and managers in Shayo et al.’s [34] study believed that involving lay health committee members in planning would lead to uninformed decisions and fail to yield desired results. Consistent with our review findings, literature supports that health professionals believe that lay community members would not grasp the complex clinical and administrative aspects of health being discussed [34–36], thus limiting the participation of communities in making decisions on matters that affected their health.

### Tokenistic participation

Tokenistic participation implies that community members are heard and allowed to argue about decisions regarding their health, but there is no guarantee that their contributions will be considered by health professionals [37]. It is widely accepted that inclusion of community members, especially marginalized groups, in making decisions about their health is crucial for them to have greater control in determining how these services are delivered. There is, however, inadequate understanding of the best way of ensuring both implicit and explicit inclusion. There is need for empirical evidence to answer the question whether inclusion will be enhanced by asking more marginalized groups to participate or whether the more influential individuals should be sensitized on how to represent marginalized groups. For example, we noted that some studies in this review only reported the number of women in health committees. Women’s contribution to decision-making was not explored in any of these studies. Existing literature shows that underlying contextual factors and culture influence the extent to which women participate in health committees. In some settings, tokenism in women’s participation in health committees is driven by androcentric and patriarchal ideologies [38, 39]. Shayo et al. [34] studied the patriarchal tendencies that limit women’s participation in health committees. First, women were not listened to and do not occupy leadership positions. This also holds a risk that women’s needs are not well represented in health committees, because they are perceived not to have gained sufficient confidence in presenting strong points during meetings. Second, male committee members were sceptical and did not trust in women’s abilities in decision making [34].. Tokenistic inclusion of women and patriarchy constrain equitable participation and gender parity in decision making structures, despite the existence of polices to promote equal and equitable gender representation.

Using the Draper et al., framework, we attempted to characterize the extent of participation in the continuum of community participation, which ranges from mobilization, collaboration to empowerment. Overall, leadership by health committees is situated on the mobilization end of the continuum because health professionals in several contexts assume the role of constituting health committees. The same health professionals did not hold “elite” members accountable whenever they contravened health committee selection guidelines when constituting health committees. On the other hand, health committees were more collaborative when voicing communities’ grievances about the quality of care to health workers. Participation of health committees in management of health services is largely in the mobilization end of the continuum. Health workers decided on the health activity plans and annual health budgets for the health centres and community health without involving health committee members. Day-to-day management and decision making about drug stocks, cleanliness and employment of support staff are in the collaborative end of the continuum of participation. Our findings suggest that resource mobilization by health committees in sub-Sahara Africa is situated in the collaborative point of the participation continuum because they mainly get their resources by seeking donations for materials and labour from their communities. They also raised funds for running their facilities by lobbying influential members in society to make financial contributions. Recent research suggests that health committees can be strengthened to effectively play their roles in leadership and management by building their capacity and providing them with regular technical support [40]. We did not find adequate empirical literature of how health committees sought to promote the participation of women in health committees. Also did not find literature on the involvement of health committees in participatory evaluation of community health programs. Generally, documentation of participatory evaluation of community level primary health programs is lacking or inadequate [41]. We interpret that both participation of women and participation of health committees in participatory evaluation are towards the mobilization end of the community participation spectrum.

### Recommendations

Our review brings out key issues that influence how health committees participate in primary and community health. We propose three recommendations for strengthening community participation through health committees. First, for health committees to be respected and legitimate representatives of their communities, the mode and procedures for selecting members must be seen to create political legitimacy and procedural justice [28]. Second, health workers and managers need supportive supervision and capacity building in team management. This may help to address fears of “relinquishing” power by allowing communities to participate in development of health plans. Health workers also need to be sensitized on the value of social inclusion and diversity in the composition of health committees. Third, it may be time to update community health strategies in sub-Sahara African countries to reflect the realities of community participation identified in this review. These updates need to acknowledge the inherent strengths of health committees and address the challenges of power and degree of participation in decision making by communities and marginalized groups.

### Limitations

This review has some limitations. Our literature search only included studies published after 2008 and only in English, it is possible that we precluded earlier in-depth explorations on community participation through health committees or studies in other languages. A key strength in this study was that selection of studies was performed by two authors. Analysis and interpretation of the findings was reviewed by three authors. Agreement on the interpretations drawn from the studies was arrived at through consensus. The Draper et al. framework was useful while extracting and interpreting data from the literature, despite data on some components of the framework being scarce. We also recognize that the Draper et al. framework does not focus on the analysis of how specific marginalized groups participate in decision-making through health committees. We recommend additional and in-depth analysis of contextual factors that contextual that influence community participation.

## Conclusion

Our study shows that health committees are viable mechanisms for community participation. However, there is need for transformational change in the practice of community participation [42]. Decision makers, health managers and advocates need to fundamentally rethink how health committees are selected, empowered and supported to implement their roles and responsibilities. There is also need to support health workers to have a more positive stand towards health committees by sensitizing them about the value of community participation in health. These insights can help health workers and decision makers to update guidelines on community participation by providing a synthesis of key issues that influence how health committees participate in health.

## Supplementary Information

**Additional file 1**: **Supplementary file 1:** Search terms applied in the three online databases. Supplementary file 1 contains all the search terms that we applied while searching for relevant articles in the MEDLINE, CINAHL and Popline data bases.

## Data Availability

All data that were analysed are included in this published article and its supplementary information files.

## Abbreviations

CHC: Community Health Committee
COSA: Comité de Santé
FGD: Focus group discussion
HCAC: Health Centre Advisory Committee
HFC: Health Facility Committee
HFCG: Health Facility Governing Committee
HMC: Health management committee
IDI: In-depth Interview
MoH: Ministry of Health,
VDC: Village Development Committee
VHT: Village Health Team
