# Supplementary material for: Participation in primary health care through community-level health committees in Sub-Saharan Africa: a qualitative synthesis
Source: BMC Public Health. 2022 Feb 19;22:359. doi: 10.1186/s12889-022-12730-y (PMC8858504; doi:10.1186/s12889-022-12730-y)
Supplement: Supplementary file 1 — Additional file 1: Supplementary file 1: Search terms applied in the three online databases. Supplementary file 1 contains all the search terms that we applied while searching for relevant articles in the MEDLINE, CINAHL and Popline data bases. [file 12889_2022_12730_MOESM1_ESM.docx]

*Table 1: Search terms applied in the three online databases*

| **Database** | **Number of articles identified** | **Search terms** |
| --- | --- | --- |
| PubMed | 257 | ((("Community Participation"[Mesh] OR public participat* OR “accountability”) AND (Health AND (facility* OR (Centre OR committee))) AND ((“health committee*” AND [community OR village]) OR (“Health facility committee*” OR “facility manage*”OR (“Health centre* committee*”) OR (“Village development committee*” AND health) OR (“Health plan*”) OR (“Health manage* committee*”) OR “facility committee” OR (“community leader*”)OR (“Health social action committee*”) OR governance OR (“Municipal health council”) OR (“Health board” AND [village OR community OR municipal]))) AND "Africa South of the Sahara"[Mesh] |
| **Popline** | 16 | ( “Health facility committee*” OR “facility manage*”OR (“Health centre* committee*”) OR (“Village development committee*” AND health) OR (“Health plan*”) OR (“Health manage* committee*”) OR “facility committee” OR (“community leader*”) OR (“Health social action committee*”) OR governance OR (“Municipal health council”) OR (“Health board” AND [village OR community OR municipal]) ) AND ( africa or sub Saharan africa or african ) AND ( Health AND (facility* OR Centre OR committee) ) AND ( "Community Participation" OR public participat* OR “accountability” ) |
| **CINAHL** | 6 |  |
